# (±)-Bis(1-carb­oxy-2-phenyl­ethanaminium) hexa­fluoro­silicate(VI)

**DOI:** 10.1107/S1600536812021587

**Published:** 2012-05-19

**Authors:** Ratiba Belhouas, Sofiane Bouacida, Chaouki Boudaren, Jean-Claude Daran, Thierry Roisnel

**Affiliations:** a Unité de Recherche de Chimie de l’Environnement et Moléculaire Structurale (CHEMS), Université Mentouri-Constantine, 25000 Algeria; b Laboratoire de Chimie de Coordination, UPR CNRS 8241, 205 route de Narbonne, 31077 Toulouse Cedex, France; c Centre de Difractométrie X, UMR 6226 CNRS Unité Sciences Chimiques de Rennes, Université de Rennes I, 263 Avenue du Général Leclerc, 35042 Rennes, France

## Abstract

The asymmetric unit of the title fluoro­silicate salt, 2C_9_H_12_NO_2_^+^·SiF_6_^2−^, consists of a phenylalaninium cation and half of a fluorosilicate anion, the Si atom being located on an inversion center. In the crystal, all of the F atoms act as hydrogen-bond acceptors and link the cations through different graph-set motifs, forming layers developing parallel to (100).

## Related literature

For applications of fluoro­silicate salts, see: Katayama *et al.* (2001 ▶); Kalem (2004 ▶); Airoldi & De Farias (2000 ▶); Han *et al.* (2000 ▶); Gelmboldt (1989 ▶); Gelmboldt *et al.* (2007 ▶). For our previous work on hydrogen-bonding inter­actions in the crystal structures of protonated amines, see: Bouacida *et al.* (2005 ▶, 2007 ▶, 2009 ▶); Benslimane *et al.* (2007 ▶); Bouacida (2008 ▶). For a description of the Cambridge Structural Database, see: Allen (2002 ▶). For hydrogen-bond motifs, see: Etter *et al.* (1990 ▶); Bernstein *et al.* (1995 ▶); Janiak (2000 ▶).
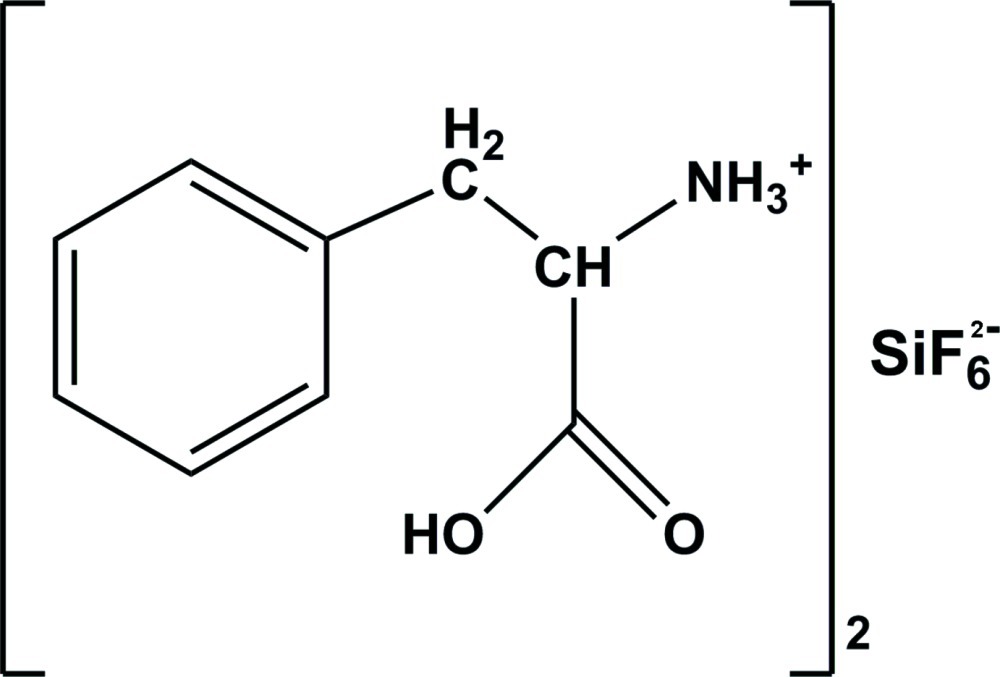


## Experimental

### 

#### Crystal data

2C_9_H_12_NO_2_^+^·SiF_6_^2−^
*M* *_r_* = 474.48
Monoclinic, 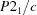

*a* = 11.183 (2) Å
*b* = 5.7531 (10) Å
*c* = 17.000 (4) Å
β = 105.59 (2)°
*V* = 1053.5 (4) Å^3^
*Z* = 2
Mo *K*α radiation
μ = 0.19 mm^−1^
*T* = 295 K
0.59 × 0.50 × 0.37 mm

#### Data collection

Nonius KappaCCD diffractometer
2412 measured reflections
2412 independent reflections
1917 reflections with *I* > 2σ(*I*)

#### Refinement

*R*[*F* ^2^ > 2σ(*F* ^2^)] = 0.035
*wR*(*F* ^2^) = 0.086
*S* = 1.03
2412 reflections
144 parameters
H-atom parameters constrained
Δρ_max_ = 0.22 e Å^−3^
Δρ_min_ = −0.19 e Å^−3^

### 

Data collection: *COLLECT* (Otwinowski & Minor, 1997 ▶); cell refinement: *DIRAX/LSQ* (Duisenberg *et al.*, 2003 ▶); data reduction: *EVALCCD* (Duisenberg *et al.*, 2003 ▶); program(s) used to solve structure: *SHELXS97* (Sheldrick, 2008 ▶); program(s) used to refine structure: *SHELXL97* (Sheldrick, 2008 ▶); molecular graphics: *ORTEPIII* (Burnett & Johnson, 1996 ▶), *ORTEP-3 for Windows* (Farrugia, 1997 ▶) and *PLATON* (Spek, 2009 ▶); software used to prepare material for publication: *WinGX* (Farrugia, 1999 ▶).

## Supplementary Material

Crystal structure: contains datablock(s) global, I. DOI: 10.1107/S1600536812021587/ez2295sup1.cif

Structure factors: contains datablock(s) I. DOI: 10.1107/S1600536812021587/ez2295Isup2.hkl

Additional supplementary materials:  crystallographic information; 3D view; checkCIF report

## Figures and Tables

**Table 1:** Hydrogen-bond geometry (Å, °)

*D*—H⋯*A*	*D*—H	H⋯*A*	*D*⋯*A*	*D*—H⋯*A*
O1—H1⋯F2^i^	0.82	1.84	2.6456 (14)	167
N2—H2*A*⋯O2^ii^	0.89	2.04	2.8511 (17)	151
N2—H2*B*⋯F3	0.89	1.88	2.7656 (15)	171
N2—H2*C*⋯F1^iii^	0.89	2.01	2.8549 (15)	158

Symmetry codes: (i) 
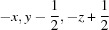
; (ii) 
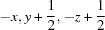
; (iii) 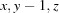.

**Fig. 1.**
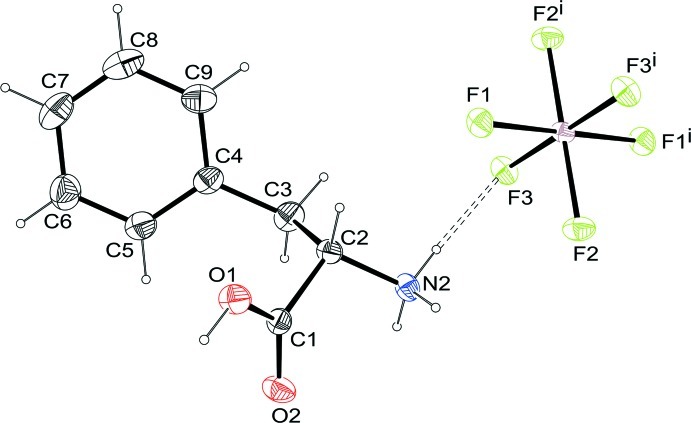
The asymmetric unit of (I) with the atom-labelling scheme. Displacement ellipsoids are drawn at the 30% probability level. H atoms are represented as small spheres of arbitrary radii. Hydrogen bond is shown as dashed line.[Symmetry codes: (i) -x, 1 - y, 1 - z]

**Fig. 2.**
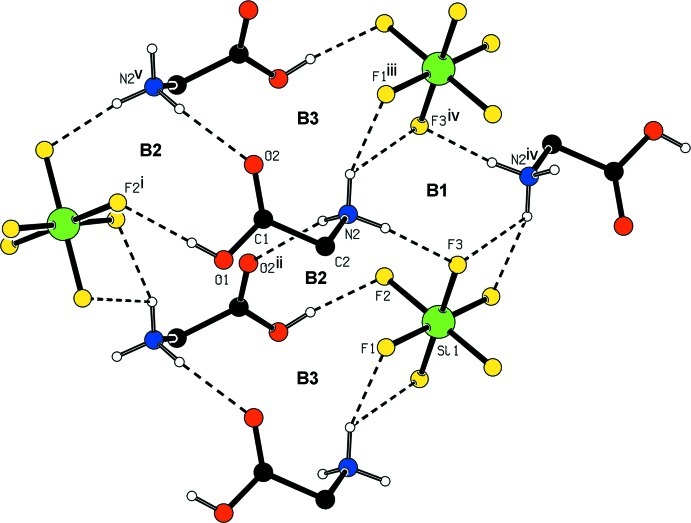
Partial view of compound (I), showing the formation of R42(8) (B1), R33(10) (B2) and R33(14) (B3) graph set motifs through N—H···F, N—H···O and O—H···F hydrogen bonds. The C6H5CH2 fragment have been omitted for the sake of clarity. [Symmetry codes: (i) -x, y - 1/2, -z + 1/2; (ii) -x, y + 1/2, -z + 1/2; (iii) x, y - 1, z; (iv) -x, -y, -z + 1; (v) -x, y - 1/2, 1/2 - z]

**Fig. 3.**
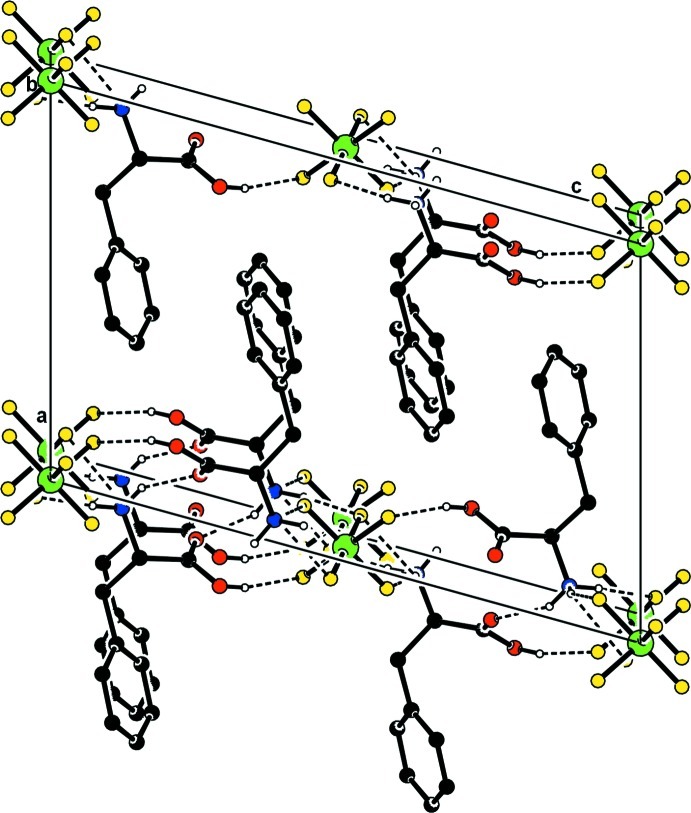
Packing view projected down the b axis showing the formation of layers parallel to the (1 0 0) plane. Hydrogen bonds are shown as dashed lines. H atoms not involved in hydrogen bonding have been omitted for clarity.

## supplementary crystallographic information

### Comment

Fluorosilicate salts involving onium cations of N– and O– containing organic bases and amino acids have practical applications as ionic liquids (Katayama *et al.*, 2001), dielectrics with cryptocrystalline structure (Kalem, 2004), layered organic-inorganic hybrid materials (Airoldi & De Farias, 2000) and chemical reagents (Han *et al.*, 2000; Gelmboldt, 1989). Their structures are commonly dominated by strong directional interactions involving F atoms and convenient hydrogen-bond donors, although the relationships in such systems can be complicated due to the presence of competitive OH and NH binding sites (Gelmboldt *et al.*, 2007). We report here the synthesis, crystal structure and hydrogen-bonded frameworks of a new hybrid compound based on fluorosilicate. The title compound (I) was prepared as part of our ongoing studies of hydrogen-bonding interactions in the crystal structures of protonated amines (Bouacida *et al.*, 2005, 2007, 2009; Benslimane *et al.*, 2007; Bouacida, 2008). From the molecular point of view, the structure is quite simple, since the individual components do not deviate from the expected geometries, with bond distances and angles lying within reported values for these species (CSD, Allen, 2002). The most attractive aspect of these structures resides in their extensive hydrogen-bonding schemes.

The asymmetric unit of (I) is built up from a (+/-)-phenylalaninium cation and half a molecule of a hexafluorosilicate anion located on an inversion center, connected by N—H···F hydrogen bonds (Fig. 1).

As observed in compound I, all the F atoms of the hexafluorosilicate anion act as hydrogen bond acceptors and are engaged in N—H···F and O—H···F bonds with the alaninium part of the cation (Fig. 2, Table 1). Two H atoms, H2B and H2C and their symmetry related counterparts (-*x*, -*y*, 1 - *z*), of the ammonium NH_3_ interact with two symmetry related fluorosilicate (-*x*, -*y*, 1 - *z*) building a *R*_4_^2^(8) ring labelled B1, whereas H2A, the third H atom of the NH_3_ and the H atom of the symmetry related (*-x*, *y* + 1/2, -*z* + 1/2) carboxylate complete a *R*_3_^3^(10) graph set motif labelled B2. Futhermore, two symmetry related (-*x*, *y* - 1/2, 1/2 - *z*) cations and one fluorosilicate (*x*, *y* - 1, *z*) form a *R*_3_^3^ (14) ring labelled B3, through N2—H2C···F1, N2—H2B···O2 and O1—H···F2 (Etter *et al.*, 1990; Bernstein *et al.*, 1995), see Table 1, Fig. 2.

These hydrogen bonds result in the formation of layers parallel to the (1 0 0) plane. In these layers, chains of cations and anions alternate (Fig. 3). As shown in the Figure, the phenyl rings of the symmetry related layers are intercalated; however the centroid to centroid distance between the phenyl rings are too long (4.958 (1) and 4.523 (1) Å) to consider π-π interactions (Janiak, 2000).

### Experimental

Crystals of compound I were grown from an aqueous solution that was obtained by dissolving 1 mmol SiO_2_ and 2 mmol phenylalanine in hydrofluoric acid (HF). The solutions were slowly evaporated to dryness for a couple of weeks. Some white crystals were carefully isolated under polarizing microscope for analysis by X-ray diffraction.

### Refinement

All non-H atoms were refined with anisotropic atomic displacement parameters. All H atoms were localized on Fourier maps but introduced in calculated positions and treated as riding on their parent C, N and O atoms, with C—H = 0.93,0.97, 0.98 Å, N—H = 0.89 Å and O—H = 0.82 Å with *U*_iso_(H) = 1.2 or 1.5 *U*_eq_(C, N or O).

### Crystal data

**Table:**

2C_9_H_12_NO_2_^+^·SiF_6_^2^^−^	*F*(000) = 492
*M**_r_* = 474.48	*D*_x_ = 1.496 Mg m^−^^3^
Monoclinic, *P*2_1_/*c*	Mo *K*α radiation, λ = 0.71073 Å
Hall symbol: -P 2ybc	Cell parameters from 4769 reflections
*a* = 11.183 (2) Å	θ = 5.0–27.5°
*b* = 5.7531 (10) Å	µ = 0.19 mm^−^^1^
*c* = 17.000 (4) Å	*T* = 295 K
β = 105.59 (2)°	Block, white
*V* = 1053.5 (4) Å^3^	0.59 × 0.50 × 0.37 mm
*Z* = 2	

### Data collection

**Table:**

Nonius KappaCCD diffractometer	1917 reflections with *I* > 2σ(*I*)
Radiation source: fine-focus sealed tube	*R*_int_ = 0.000
Graphite monochromator	θ_max_ = 27.5°, θ_min_ = 5.0°
Detector resolution: 9 pixels mm^-1^	*h* = −14→13
CCD rotation images, thick slices scans	*k* = 0→7
2412 measured reflections	*l* = 0→22
2412 independent reflections	

### Refinement

**Table:**

Refinement on *F*^2^	Primary atom site location: structure-invariant direct methods
Least-squares matrix: full	Secondary atom site location: difference Fourier map
*R*[*F*^2^ > 2σ(*F*^2^)] = 0.035	Hydrogen site location: inferred from neighbouring sites
*wR*(*F*^2^) = 0.086	H-atom parameters constrained
*S* = 1.03	*w* = 1/[σ^2^(*F*_o_^2^) + (0.0329*P*)^2^ + 0.2833*P*] where *P* = (*F*_o_^2^ + 2*F*_c_^2^)/3
2412 reflections	(Δ/σ)_max_ = 0.001
144 parameters	Δρ_max_ = 0.22 e Å^−^^3^
0 restraints	Δρ_min_ = −0.19 e Å^−^^3^

### Special details

**Table:**

Geometry. All e.s.d.'s (except the e.s.d. in the dihedral angle between two l.s. planes) are estimated using the full covariance matrix. The cell e.s.d.'s are taken into account individually in the estimation of e.s.d.'s in distances, angles and torsion angles; correlations between e.s.d.'s in cell parameters are only used when they are defined by crystal symmetry. An approximate (isotropic) treatment of cell e.s.d.'s is used for estimating e.s.d.'s involving l.s. planes.
Refinement. Refinement of *F*^2^ against ALL reflections. The weighted *R*-factor *wR* and goodness of fit *S* are based on *F*^2^, conventional *R*-factors *R* are based on *F*, with *F* set to zero for negative *F*^2^. The threshold expression of *F*^2^ > σ(*F*^2^) is used only for calculating *R*-factors(gt) *etc*. and is not relevant to the choice of reflections for refinement. *R*-factors based on *F*^2^ are statistically about twice as large as those based on *F*, and *R*- factors based on ALL data will be even larger.

### Fractional atomic coordinates and isotropic or equivalent isotropic displacement parameters (Å^2^)

**Table:**

	*x*	*y*	*z*	*U*_iso_*/*U*_eq_	
Si1	0.0000	0.5000	0.5000	0.02345 (13)	
F1	0.07812 (8)	0.54094 (14)	0.43002 (5)	0.0345 (2)	
F2	−0.11419 (8)	0.36774 (15)	0.42781 (5)	0.0371 (2)	
F3	0.07100 (8)	0.24091 (13)	0.52540 (5)	0.0346 (2)	
O1	0.17794 (11)	0.0905 (2)	0.21347 (6)	0.0403 (3)	
H1	0.1700	0.0176	0.1709	0.060*	
O2	0.10064 (12)	−0.23340 (19)	0.25348 (6)	0.0431 (3)	
N2	0.05617 (12)	0.0158 (2)	0.37935 (7)	0.0307 (3)	
H2A	−0.0126	0.0728	0.3455	0.046*	
H2B	0.0661	0.0763	0.4289	0.046*	
H2C	0.0499	−0.1381	0.3820	0.046*	
C2	0.16410 (14)	0.0759 (2)	0.34897 (8)	0.0293 (3)	
H2	0.1696	0.2449	0.3435	0.035*	
C1	0.14352 (13)	−0.0392 (2)	0.26636 (8)	0.0289 (3)	
C3	0.28085 (15)	−0.0161 (3)	0.41120 (9)	0.0410 (4)	
H3A	0.2872	0.0580	0.4634	0.049*	
H3B	0.2708	−0.1816	0.4182	0.049*	
C4	0.40009 (15)	0.0225 (3)	0.38861 (9)	0.0374 (4)	
C6	0.55760 (19)	−0.1169 (4)	0.32857 (12)	0.0563 (5)	
H6	0.5865	−0.2308	0.2994	0.068*	
C9	0.47022 (18)	0.2226 (3)	0.41237 (11)	0.0493 (4)	
H9	0.4405	0.3395	0.4399	0.059*	
C8	0.58322 (19)	0.2497 (4)	0.39562 (12)	0.0585 (5)	
H8	0.6297	0.3835	0.4124	0.070*	
C5	0.44501 (17)	−0.1453 (3)	0.34602 (11)	0.0469 (4)	
H5	0.3988	−0.2791	0.3289	0.056*	
C7	0.62729 (18)	0.0799 (4)	0.35423 (12)	0.0580 (5)	
H7	0.7041	0.0975	0.3435	0.070*	

### Atomic displacement parameters (Å^2^)

**Table:**

	*U*^11^	*U*^22^	*U*^33^	*U*^12^	*U*^13^	*U*^23^
Si1	0.0314 (3)	0.0221 (2)	0.0178 (2)	−0.0039 (2)	0.00823 (19)	−0.00049 (18)
F1	0.0451 (5)	0.0334 (4)	0.0310 (4)	−0.0022 (4)	0.0206 (4)	0.0025 (3)
F2	0.0414 (5)	0.0393 (5)	0.0281 (4)	−0.0112 (4)	0.0049 (4)	−0.0044 (3)
F3	0.0472 (5)	0.0277 (4)	0.0311 (4)	0.0050 (4)	0.0141 (4)	0.0041 (3)
O1	0.0516 (7)	0.0483 (6)	0.0232 (5)	−0.0117 (5)	0.0137 (5)	−0.0002 (4)
O2	0.0606 (8)	0.0396 (6)	0.0310 (5)	−0.0123 (5)	0.0156 (5)	−0.0066 (5)
N2	0.0388 (7)	0.0284 (6)	0.0283 (6)	0.0025 (5)	0.0147 (5)	0.0024 (5)
C2	0.0359 (8)	0.0285 (7)	0.0252 (6)	−0.0043 (6)	0.0110 (6)	−0.0002 (5)
C1	0.0274 (7)	0.0352 (8)	0.0238 (6)	0.0002 (6)	0.0065 (5)	0.0010 (5)
C3	0.0388 (9)	0.0543 (10)	0.0265 (7)	−0.0026 (7)	0.0031 (6)	0.0038 (7)
C4	0.0324 (8)	0.0468 (9)	0.0280 (7)	−0.0026 (7)	−0.0003 (6)	0.0029 (6)
C6	0.0453 (11)	0.0653 (12)	0.0589 (11)	0.0071 (9)	0.0150 (9)	−0.0007 (9)
C9	0.0484 (11)	0.0497 (10)	0.0441 (9)	−0.0052 (8)	0.0027 (8)	−0.0041 (8)
C8	0.0491 (12)	0.0588 (12)	0.0586 (11)	−0.0176 (10)	−0.0009 (9)	0.0077 (10)
C5	0.0412 (10)	0.0472 (10)	0.0502 (10)	−0.0055 (8)	0.0088 (8)	−0.0044 (7)
C7	0.0349 (10)	0.0765 (14)	0.0596 (11)	−0.0056 (10)	0.0071 (9)	0.0138 (10)

### Geometric parameters (Å, º)

**Table:**

Si1—F1^i^	1.6711 (9)	C2—H2	0.9800
Si1—F1	1.6711 (9)	C3—C4	1.500 (2)
Si1—F3^i^	1.6895 (8)	C3—H3A	0.9700
Si1—F3	1.6895 (8)	C3—H3B	0.9700
Si1—F2	1.6952 (8)	C4—C5	1.380 (3)
Si1—F2^i^	1.6952 (9)	C4—C9	1.391 (2)
O1—C1	1.3036 (17)	C6—C7	1.377 (3)
O1—H1	0.8200	C6—C5	1.379 (3)
O2—C1	1.2121 (17)	C6—H6	0.9300
N2—C2	1.4760 (18)	C9—C8	1.377 (3)
N2—H2A	0.8900	C9—H9	0.9300
N2—H2B	0.8900	C8—C7	1.371 (3)
N2—H2C	0.8900	C8—H8	0.9300
C2—C1	1.5135 (19)	C5—H5	0.9300
C2—C3	1.537 (2)	C7—H7	0.9300
			
F1^i^—Si1—F1	180.0	O2—C1—O1	125.37 (13)
F1^i^—Si1—F3^i^	90.38 (4)	O2—C1—C2	121.67 (13)
F1—Si1—F3^i^	89.62 (4)	O1—C1—C2	112.95 (12)
F1^i^—Si1—F3	89.62 (4)	C4—C3—C2	114.97 (12)
F1—Si1—F3	90.38 (4)	C4—C3—H3A	108.5
F3^i^—Si1—F3	180.0	C2—C3—H3A	108.5
F1^i^—Si1—F2	90.90 (5)	C4—C3—H3B	108.5
F1—Si1—F2	89.10 (4)	C2—C3—H3B	108.5
F3^i^—Si1—F2	90.00 (4)	H3A—C3—H3B	107.5
F3—Si1—F2	90.00 (4)	C5—C4—C9	118.37 (17)
F1^i^—Si1—F2^i^	89.10 (4)	C5—C4—C3	120.33 (15)
F1—Si1—F2^i^	90.90 (5)	C9—C4—C3	121.26 (16)
F3^i^—Si1—F2^i^	90.00 (4)	C7—C6—C5	120.05 (19)
F3—Si1—F2^i^	90.00 (4)	C7—C6—H6	120.0
F2—Si1—F2^i^	180.0	C5—C6—H6	120.0
C1—O1—H1	109.5	C8—C9—C4	120.73 (18)
C2—N2—H2A	109.5	C8—C9—H9	119.6
C2—N2—H2B	109.5	C4—C9—H9	119.6
H2A—N2—H2B	109.5	C7—C8—C9	120.13 (18)
C2—N2—H2C	109.5	C7—C8—H8	119.9
H2A—N2—H2C	109.5	C9—C8—H8	119.9
H2B—N2—H2C	109.5	C6—C5—C4	120.84 (17)
N2—C2—C1	106.69 (11)	C6—C5—H5	119.6
N2—C2—C3	107.57 (11)	C4—C5—H5	119.6
C1—C2—C3	112.16 (13)	C8—C7—C6	119.84 (19)
N2—C2—H2	110.1	C8—C7—H7	120.1
C1—C2—H2	110.1	C6—C7—H7	120.1
C3—C2—H2	110.1		
			
N2—C2—C1—O2	−39.96 (18)	C4—C9—C8—C7	0.8 (3)
C3—C2—C1—O2	77.58 (18)	C7—C6—C5—C4	0.5 (3)
N2—C2—C1—O1	140.88 (13)	C9—C4—C5—C6	1.0 (2)
C3—C2—C1—O1	−101.58 (15)	C3—C4—C5—C6	−176.65 (15)
N2—C2—C3—C4	177.95 (13)	C9—C8—C7—C6	0.8 (3)
C1—C2—C3—C4	60.93 (18)	C5—C6—C7—C8	−1.4 (3)
C2—C3—C4—C5	−92.09 (19)	C4—C3—C2—C1	60.93 (18)
C2—C3—C4—C9	90.30 (18)	C4—C3—C2—N2	177.95 (13)
C5—C4—C9—C8	−1.7 (2)	C3—C2—C1—O1	−101.58 (15)
C3—C4—C9—C8	175.98 (15)	C3—C2—C1—O2	77.58 (18)

Symmetry code: (i) −*x*, −*y*+1, −*z*+1.

### Hydrogen-bond geometry (Å, º)

**Table:**

*D*—H···*A*	*D*—H	H···*A*	*D*···*A*	*D*—H···*A*
O1—H1···F2^ii^	0.82	1.84	2.6456 (14)	167
N2—H2*A*···O2^iii^	0.89	2.04	2.8511 (17)	151
N2—H2*B*···F3	0.89	1.88	2.7656 (15)	171
N2—H2*C*···F1^iv^	0.89	2.01	2.8549 (15)	158

Symmetry codes: (ii) −*x*, *y*−1/2, −*z*+1/2; (iii) −*x*, *y*+1/2, −*z*+1/2; (iv) *x*, *y*−1, *z*.
